# Background predictors of time to death in infancy: evidence from a survival analysis of the 2018 Nigeria DHS data

**DOI:** 10.1186/s12889-021-12424-x

**Published:** 2022-01-06

**Authors:** Michael Kunnuji, Idongesit Eshiet, Bright Opoku Ahinkorah, Temitope Omogbemi, Sanni Yaya

**Affiliations:** 1 Department of Sociology, University of Lagos, Lagos, Nigeria; 2 School of Public Health, University of Technology Sydney, Sydney, Australia; 3 School of International Development and Global Studies, Faculty of Social Sciences, University of Ottawa, 120 University Private, Ottawa, ON K1N 6N5 Canada; 4 The George Institute for Global Health, Imperial College London, London, UK

**Keywords:** Time to death in infancy, Survival analysis, Nigeria: DHS, Global Health

## Abstract

**Background:**

Nigeria’s child health profile is quite concerning with an infant mortality rate of 67 deaths per 1000 live births and a significant slowing down in progress towards improving child health outcomes. Nigeria’s 2018 Demographic and Health Survey (DHS) suggests several bio-demographic risk factors for child death, including mother’s poor education, poverty, sex of child, age of mother, and location (rural vs urban) but studies are yet to explore the predictive power of these variables on infant survival in Nigeria.

**Methods:**

The study extracted data for all births in the last 12 months preceding the 2018 Nigeria DHS and used the Cox proportional hazard model to predict infant survival in Nigeria. Failure in this analysis is death with two possible outcomes – dead/alive – while the survival time variable is age at death. We censored infants who were alive at the time of the study on the day of the interview. Covariates in the analysis were: age of mother, education of mother, wealth quintile, sex of child, location, region, place of delivery, and age of pregnancy.

**Results:**

The study found that a higher education of a mother compared to no education (*β =* .429; *p*-value < 0.05); belonging to a household in the richer wealth quintile (*β =* .618; *p*-value < 0.05) or the highest quintile (*β =* .553; *p*-value < 0.05), compared to the lowest wealth quintile; and living in North West (*β =* 1.418; *p*-value < 0.05) or South East zone (*β =* 1.711; *p*-value < 0.05), significantly predict infant survival.

**Conclusion:**

Addressing Nigeria’s infant survival problem requires interventions that give attention to the key drivers – education, socio-economic status, and socio-cultural contextual issues. We therefore recommend full implementation of the universal basic education policy, and child health education programs targeted at mothers as long- and short-term solutions to the problem of poor child health outcomes in Nigeria. We also argue in favor of better use of evidence in policy and program development in Nigeria.

## Background

Globally, the past two decades have been marked by significant decline in neonatal, infant and under-five mortality rates [1]. Recent Demographic and Health Surveys (DHS) in Nigeria show that the decline in child mortality rates is slowing down. While the country recorded a decline from 201 under-five deaths per 1000 live births in 2003 to 157 in 2008 and 128 in 2013, the rate rose slightly to 132 deaths per 1000 live births in 2018. A similar trend was recorded in neonatal mortality rate which declined from 48 deaths per 1000 live births in 2003 to 40 in 2008 and 37 in 2013 only to rise slightly to 39 in 2018. Significant gains were also recorded in infant mortality rates which declined from 100 deaths per 1000 live births to 75 deaths between 2003 and 2013. It further declined to 69 in 2013, but between 2013 and 2018, infant mortality barely declined, with a 2018 rate of 67 deaths per 1000 live births [2].

At present, more than half a million Nigerian infants die yearly (United Nations Inter-Agency Group for Child Mortality Estimation (UN IGME), 2019) and achieving the Sustainable Development Goal (SDG 3) target of reducing neonatal mortality rate and under-five mortality rate to 12 and 25 deaths per 1000 live births respectively by 2030 [3] may be unattainable on current trends. Repositioning Nigeria on the path of steady and sustained child mortality decline requires applying strong scientific evidence in revising the country’s child health policy and programs. Nationally representative surveys in Nigeria have consistently shown subnational variation and biodemographic factors by which rates vary significantly among sub-populations [2, 4]. If this variation is sufficiently understood, programs based on such evidence may yield better results as they would be designed to target sub-populations with the most needs.

The DHS reports show some ‘biodemographic risk factors’ for child mortality but there is need for analysis using models that allow for variable control. Mother’s education, wealth, sex of child, age of mother, and location (rural vs urban) are associated with the death/survival of a child [2]. This study builds on this to show the predictors of child death in infancy when hazard ratios are adjusted in a model with multiple covariates. It is known, for instance, that education and wealth are significant predictors of several variables such as location and place of delivery which may influence child survival [2]. What we do not know yet is whether location and place of delivery are predictors of child survival if education and wealth are held constant using recent nationally representative data. In addition, do variables such as the sex of a child, a mother’s age, duration of pregnancy and region predict the occurrence of death in infancy among Nigerian children? Answering these questions holds much for effective programming to reduce infant mortality and improve Nigeria’s child health policy.

Proximate predictors of child deaths include socio-demographic factors [5–8], biomedical factors such as birth interval, weight and gestation period, sex of child and diseases [9–16] and health-seeking behavior and barriers to care [17, 18]. Research shows that education, income, and access to healthcare resources (including safe drinking water and sanitation) enhance child survival globally [5–8, 10, 15, 19, 20], and countries having higher inequality in access to education, income and healthcare resources also have higher mortality rates [5, 8]. Other predictors include region/geographic inequalities [19–22], age of mother (at first birth/current) [20, 21] and health-seeking behavior for children, with the use of facility-based care for delivery and child illness leading to improved child survival [22–25]. Use of facility-based care is itself influenced by factors like location, education, socioeconomic status, and socio-cultural context [25–30].

In summary, previous studies show that different categories of nonbiomedical and biomedical predictors of child death may exist, including socio-demographic factors such as wealth, education, location, age of mother, sex of child, gestational period, and health seeking behavior. This study’s primary objective is to show how mother’s education, household poverty, sex of child, age of mother, and location (whether rural or urban), place of delivery and whether pregnancy is pre- or full-term predict the survival of infants in Nigeria. It is important to know whether these factors continue to predict child survival even as infant mortality continues to decline slowly. While the DHS report implies that sex of child, age of mother and location (rural vs urban) are predictors of child survival, this study seeks to show whether these variables retain their predictive power in a survival analysis of infants with multiple covariates. It is possible for infant mortality to appear to be associated with or predicted by single variables when not controlling for confounding effects of other variables. For instance, living in a rural area appears to be a significant predictor of infant death in the DHS report with an infant mortality rate of 56 deaths per 1000 live births, compared to a rate of 74 deaths per 1000 live births in urban settlements. Other variables such as mother’s education and household wealth may be responsible for this observation and living in a rural area may not increase the risk of infant death. The current study addresses this problem.

## Methods

### Data source

The data used for this study came from the 2018 Nigeria DHS which was implemented by the National Population Commission [Nigeria] with technical support from ICF International through the DHS Program, between August and December 2018. The DHS used Nigeria’s most recent census data as a sampling frame to achieve a nationally representative sample of women 15–49 years. The study adopted a stratified sampling technique which identified two strata – rural and urban – in each of Nigeria’s 36 states and the Federal Capital Territory, resulting in a total of 74 strata. A total of approximately 42,000 households were selected for the study and all eligible women (resident or stayed in the household the night preceding the interview) in selected households were included in the study. A total of 41,821 women were interviewed. The DHS questionnaire, which has a section on birth history and child mortality, was administered in the local languages – Hausa, Igbo, and Yoruba – by specially trained data collectors using computer-assisted personal interviewing devices [2]. The birth data file contains 127,545 cases from which 7700 births that occurred within 12 months prior to the DHS interviews were selected for analysis.

### Data extraction and selection of variables

The failure event in the analysis is death while the survival time is the age of infant in months at the time of death. Living children were right censored at the time of the interview. The independent variables in the study are mother’s education, wealth quintile, place of residence (urban vs rural), region, mother’s age, child’s sex, duration of pregnancy and place of birth. Mother’s education was recoded into four categories. ‘Some primary education’ and ‘completed primary education’ were merged, and ‘some secondary education’ and ‘completed secondary education’ categories were merged. Mother’s age was recoded into three categories – teenage mothers (< 20 years), 20–39 years and 40–49 years. The duration of pregnancy was recoded into pre-term (less than 37 weeks) and full term (37 weeks or more). We recoded place of delivery into two categories – facility delivery (government hospitals, health centers, health posts and other public facilities, private hospitals, and clinics); and home delivery (which includes mother’s homes, and other places (representing 1.47% of all cases) such as spiritual and traditional birth attendants’ places. All other variables were used in the form which they originally appeared in the DHS data.

### Statistical analysis

Univariate analysis was carried out for the socio-demographic characteristics and child survival. Next, we used Pearson’s chi-square test of independence to determine bivariate associations between child survival and the socio-demographic characteristics. Finally, we employed the Cox proportional hazards model which models time to a failure (death) using the formula:

$$(h)\left(\mathrm{t}\right)={h}_0(t)\exp \Big({\beta}_1{x}_1+\cdots +{\beta}_k{x}_k$$

where: *h*(t) represents the hazard of a failure (death); *h*_0_ represents the baseline hazard; and *β*_k_ represents the coefficients of the covariates – mother’s education, wealth quintile, age, region, location, sex of child, duration of pregnancy, and place of delivery. The analysis includes both unadjusted and adjusted hazard ratios at 95% confidence intervals (CI). For all the covariates, the first categories were used as the reference categories. The analysis was done with Stata Version 16.

### Ethics approvals

Ethics approval was not required for this study since the data is secondary and is available in the public domain. More details regarding DHS data and ethical standards are available at: http://goo.gl/ny8T6X.

## Results

A little more than half (51%) of the births were male births. Only 1% of the births were pre-term births (less than 37 weeks). More than half of the births (53%) happened in mothers’ homes. Births in government health centers, government hospitals and private hospitals/clinics represented 14, 13 and 12%, respectively. The ages of mothers of children included in the study ranged from 15 to 49 years, with a mean of 27.9 years (SD = 6.8 years). About 45% of the mothers had no formal education, and almost a third (32.8%) had secondary education while 7% had higher education. Most of the births occurred in the northern geopolitical zones with the North Central, Northeast and Northwest accounting for 18, 21 and 32%, respectively; and the Southeast, South South and Southwest accounting for 11, 9 and 10%, respectively. About two-thirds (67%) of the births occurred in rural areas, and women in lower wealth quintiles accounted for most of the births, the lowest, second and third quintiles accounting for 23, 24 and 22% of the births, respectively. About 12% of the children died.

As presented in Table 1, about three of every five infant deaths (59%) occurred before the first month, typically around the time of birth. Socio-demographic variables associated with dying included mother’s education (χ^2^ = 35.3001; *P* = 0.000); wealth quintile (χ^2^ = 37.4980; *P* < 0.000); location (χ^2^ = 18.7750; P = 0.000); duration of pregnancy (χ^2^ = 213.5547; *P* < 0.001); and place of delivery (χ^2^ = 9.0915; *P* = 0.003). Child death was associated with low education, being poor, living in a rural community, pre-term birth and home delivery.

**Table 1:** Child survival by socio-demographic characteristics

	Child alive	Child dead	Total	χ^2^; *p*-values
**Age of mother**				χ^2^ = 0.6859; *p* = 0.710
15–19 years	573 (88.15)	77 (11.85)	650 (100)	
20–39 years	5776 (87.8)	804 (12.2)	6580 (100)	
40–49 years	407 (86.6)	63 (13.4)	470 (100)	
**Education**				χ^2^ = 35.3001; *p* < 0.001
No formal education	2962 (85.8)	492 (14.2)	3454 (100)	
Completed/some primary education	1007 (86.6)	156 (13.4)	1163 (100)	
Completed/some secondary education	2273 (90.1)	249 (9.9)	2522 (100)	
Higher education	514 (91.6)	47 (8.4)	561 (100)	
**Wealth**				χ^2^ = 37.4980; *p* < 0.001
Lowest quintile	1506 (85.4)	257 (14.6)	1763 (100)	
Second quintile	1586 (86.2)	254 (13.8)	1840 (100)	
Third quintile	1441 (87.2)	212 (12.8)	1653 (100)	
Fourth quintile	1279 (90.5)	134 (9.5)	1413 (100)	
Highest quintile	944 (91.6)	87 (8.4)	1031 (100)	
**Region**				χ^2^ = 47.0126; *p* < 0.001
North Central	1198 (88.8)	151 (11.2)	1349 (100)	
Northeast	1407 (86.5)	220 (13.5)	1627 (100)	
Northwest	2074 (84.9)	369 (15.1)	2443 (100)	
Southeast	753 (90.0)	84 (10.0)	837 (100)	
South South	648 (91.8)	58 (8.2)	706 (100)	
Southwest	676 (91.6)	62 (8.4)	738 (100)	
**Place of residence**				χ^2^ = 18.7750; *p* < 0.001
Urban	2289 (90.1)	253 (9.95)	2542 (100)	
Rural	4467 (86.6)	691 (13.4)	5158 (100)	
**Sex of child**				χ^2^ = 2.3971; *p* = 0.122
Male	3411 (87.2)	502 (12.8)	3913 (100)	
Female	3345 (88.3)	442 (11.7)	3787 (100)	
**Duration of pregnancy**				χ^2^ = 213.5547; *p* < 0.001
Pre-term	44 (41.5)	62 (58.5)	106 (100)	
Full term	6712 (88.4)	882 (11.6)	7594 (100)	
**Place of delivery**				χ^2^ = 9.0915; *P* = 0.003
Home and others	4041 (86.8	613 (13.2)	4654 (100)	
Health facility	2715 (89.1)	331 (10.9)	3046 (100)	
Total	6756 (87.7)	944 (12.3)	7700 (100)	

**Fig. 1:**
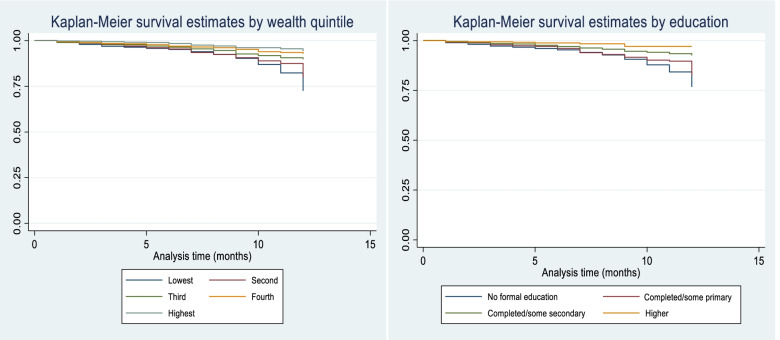
Kaplan-Meier survival estimates by wealth and education

A major predictor of the occurrence of failure (death) in infants is education when other variables in the model are controlled as Table 2 shows. While children born by mothers with primary and secondary education do not differ significantly from those without formal education in likelihood of failure, infant death adjusted hazard ratios are significantly lower for children of mothers with higher education (aHR *=* .429; *p*-value < 0.05). Table 2 further shows significantly lower adjusted hazard ratios for children in the fourth quintile (aHR *=* .618; *p*-value < 0.05) and the highest quintile (aHR *=* .553; p-value < 0.05). Infants in the Northwest have a significantly higher adjusted hazard ratio (aHR *=* 1.418; p-value < 0.05) than those in the North Central (the reference category) when all the variables in the model are controlled. The analysis further shows that when key predictors of infant death (especially education and wealth) are controlled, infants in the Southeast have a higher adjusted hazard ratio than those in the North Central. Living in a rural community or having a home delivery does not significantly affect the adjusted hazard ratio for infant death when education, wealth and region are controlled. Figure 1 shows the Kaplan-Meier survival estimates for wealth and education.

**Table 2:** Cox regression model for infant death hazard ratios

Background variables	uHR (95% CI)	*p*-value	aHR* (95% CI)	*p*-value
*Education*				
No education (RC)	1	.	1	
Primary education	.78 (0.59–1.03)	.084	1.0 (.74–1.36)	0.993
Secondary education	.46 (0.35–0.58)	.000	.72 (0.52–1.01)	0.06
Higher education	.21 (0.10–0.42)	.000	.43 (0.20–0.94)	0.35
*Wealth quintile*				
Lowest quintile (RC)	1	.	1	
Second quintile	.82 (0.64–1.05)	.113	.90 (0.70–1.16)	0.405
Third quintile	.57 (0.42–0.75)	.000	.74 (0.54–1.02)	0.063
Fourth quintile	.40 (0.28–0.55)	.000	.62 (0.42–0.92)	0.018
Highest quintile	.26 (0.17–0.41)	.000	.55 (0.32–0.96)	0.036
*Place of residence*				
Urban	1	.	1	
Rural	1.78 (1.40–2.27)	.000	1.19 (0.90–1.57)	0.232
*Age of mother*				
15–19 years (RC)	1	.	1	
20–39 years	1.01 (0.69–1.48)	.966	1.20 (0.81–1.77)	0.36
40–49 years	1.29 (0.78–2.12)	.319	1.34 (0.81–2.23)	0.261
*Sex of child*				
Male (RC)	1	.	1	
Female	1.00 (0.82–1.22)	.985	.98 (0.81–1.20)	0.868
*Duration of pregnancy*				
Pre-term (RC)	1	.	1	
Full term	.73 (0.27–1.96)	.535	.52 (0.19–1.39)	0.191
*Place of delivery*				
Home delivery (RC)	1	.	1	
Facility delivery	.51 (0.41–0.64)	.000	.82 (0.62–1.08)	0.149
*Region*				
North Central (RC)	1	.	1	
Northeast	1.69 (1.20–2.38)	0.002	1.31 (0.92–1.86)	0.133
Northwest	1.89 (1.38–2.60)	0.000	1.42 (1.01–1.99)	0.042
Southeast	1.19 (0.78–1.82)	.423	1.71 (1.09–2.69)	0.02
South South	.91 (0.57–1.48)	.712	1.12 (0.69–1.84)	0.645
Southwest	.53 (0.29–0.95)	.034	.74 (0.41–1.36)	0.333

*uHR* Unadjusted Ratio, *aHR* Adjusted Hazard Ratio, *Model Chi-square* 99.653, *p*-value 0.000, *Proportional hazards assumption test chi square* 18.21 (*p*-value = 0.1495). ^*^ Model adjusted for education and wealth

## Discussion

Poverty, region, and poor education are the major drivers of infant mortality in Nigeria in agreement with several other studies [5, 8, 15, 19]. Poverty is a factor because parents who cannot afford quality care are likely to delay use of facility-based care in the hope that their infants’ health will improve with the use of traditional medicine or care with drugs purchased from Patent Medicine Vendors [29]. Seeking facility-based care often comes with the costs of transportation, hospital registration, medical tests, and drugs which caregivers may not be able to afford. Child health services are generally poorly funded with a significant proportion of funding coming from donor agencies, NGOs and private citizens [31]. Despite Nigeria’s adoption of the Free Maternal and Child Health Care Programme, inadequate manpower and poor funding (resulting in drug stuck out, poor remuneration of workers and poor infrastructure) remains major challenges to its implementation [32]. In many rural areas where most of Nigeria’s poor people are located [33] for instance, the primary healthcare centers are not functional and caregivers are often referred to higher levels of care outside their communities, further leading to additional spending on transportation and delay in reaching care [34]. For many poor households, providing care for sick infants may also be affected by their economic activities which may not be as flexible as those of richer households because the poor have little control over their work schedule and staying away from work may imply loss of income [34].

On the other hand, having little education has negative implications for caregivers’ knowledge of health conditions of infants and what may be a major health risk. Education may also reduce acceptance of myths and unhealthy traditional practices as well as increase acceptance of health-promoting practices such as a high level of hygiene and sanitation in caregivers [30]. An interesting unanticipated finding from this study is that region is a predictor of infant survival when the influence of other variables in the model are controlled. Specifically, Southeast infants have a high risk of dying in infancy when the effect of education and wealth are controlled. This finding is not obvious in the DHS report which simply presents the infant mortality rates for the regions. Going by that report, the Southeast zone has one of the lowest rates (48 deaths per 1000 live births) in the country. Only the Southwest has a ‘lower rate’ of 43 deaths per 1000 live births [2]. Our analysis shows that there may be several factors in some regions driving infant mortality outside the variables documented by the DHS. While education and wealth may be having strong influence on child health outcomes, context specific factors such as traditional practices and health beliefs may be impacting child health negatively especially among the poor and poorly educated population of caregivers, mostly in the rural parts of the country.

An interesting finding of this study is that major biodemographic factors such as sex of child, age of mother, whether pregnancy is carried to term or delivered pre-term, and place of delivery do not significantly predict infant survival when household wealth, region and education are considered. By implication, the sex of a child, the mother’s age, pre-term birth and giving birth at home do not matter much in child survival if other conditions – having access to financial resources, having a higher education, and not living in the Northwest or Southeast – are right. This contradicts some previous studies [9–16] that concluded that these variables matter in child survival. On the strength of our analysis, we argue that a pre-term birth stands a good survival chance if the socio-cultural context is right, if resources are available to provide quality healthcare and nourishment for the child and the mother has higher education. In the same vein, these three factors (i.e., wealth, education, and region) have the potential of taking away differentials in the survival of male and female infants, children born in health facilities and at home, and children born to adolescent, adult, and older mothers.

### Strengths and limitations of the study

**Fig. 2:**
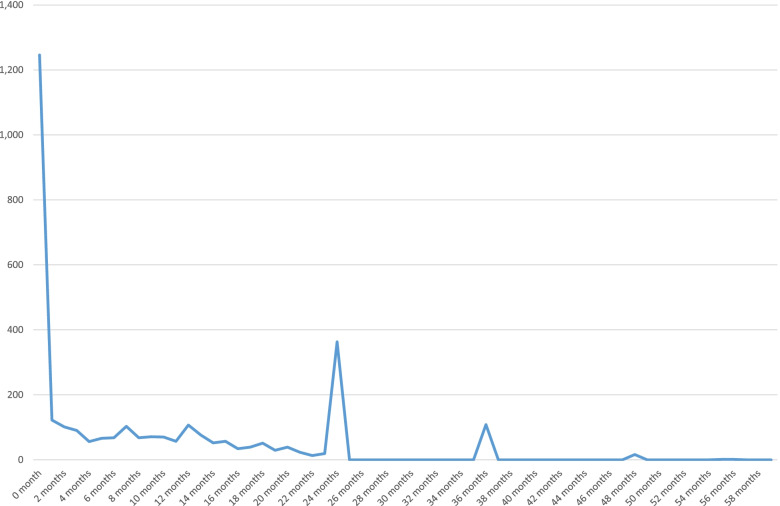
Reported ages (in month) of under-five children at death

The major strength of the study is the use of a large sample from a nationally representative survey data. Another strength is the complex statistical analysis carried out in the study, which gives room for controlling the effect of confounders and allows for greater reliability of the findings. Despite these strengths, the study also has some limitations that need to be considered. Maternal malaria, maternal anemia, maternal working status, father’s education, father’s working status, ANC attendance, iron supplementation adherence, birth weight are all potential contributors to neonatal mortality that were not explored in this study. While these are important variables with possible confounding effects, they are outside the scope of the present study. In addition, the data on time of death may not be accurate to the nearest month beyond the first year. For most children who died between their first birthday and 59 months, age at death was approximated to the nearest year (12 months), making survival analysis beyond the first year difficult or unreliable. Reported ages at death in Fig. 2 suggests heaping around 24 months, 36 months, and 48 months. With this heaping, the answers provided about time to death are not accurate and cannot be relied on for survival analysis. This analysis was originally designed to model under-five child survival but was limited to infant survival because of the said lack of precision in documented age at death. An analysis of predictors of child death beyond the first birthday will help address issues in child death which may differ from those implicated in infant death. The reliability of the information provided in this article may also be limited by caregivers’ poor recall of events.

## Conclusion

The key drivers of infant mortality in Nigeria are low level of education, poverty, and underlying region specific socio-cultural/contextual factors at play in the Northern regions and surprisingly, the Southeast. Addressing the problem requires massive investment in education and women empowerment. Nigeria’s basic education policy needs to be better pursued to ensure that the first 9 years of education is truly free and compulsory and education beyond this level should equally be encouraged. Although this is a long-term solution, it is the most promising solution to the problems of infant and child mortality in Nigeria. Economic empowerment is also a requirement for addressing Nigeria’s infant mortality problem. Strengthening child health education for caregivers through healthcare facilities and introducing nationwide programs to provide useful information to caregivers through innovative strategies for reaching them in informal settings are equally promising strategies to addressing Nigeria’s child health mortality challenge. This is because more than half of mothers do not use formal care and cannot be reached through health facilities as this study has shown.

As suggested previously, there is need to target policymakers with advocacy and public health education to improve their commitment to maternal and child healthcare in Nigeria [35]. The child health policy environment in Nigeria does not suggest sufficient commitment on the part of key actors. Policymakers and programmers also need to better use evidence to improve health policies in Nigeria [36]. Programs should seek to institute focused analysis of data from studies such as the DHS, the Verbal and Social Autopsy, and the Multiple Indicator Cluster Survey for improved programming and policymaking. In the same vein, there is need for more Maternal, Newborn and Child Health intervention research [37] for improved health outcomes.

## Data Availability

Data for this study were sourced from Demographic and Health surveys (DHS) and available here: http://dhsprogram.com/data/available-datasets.cfm.

## Abbreviations

aHR: Adjusted hazard ratios
CI: Confidence interval
DHS: Demographic and Health Surveys
SDGs: Sustainable Development Goals
uHR: Unadjusted Ratio
